# From biological scaffold to multifunctional bioplatform: research progress and applications of decellularized extracellular matrix

**DOI:** 10.3389/fbioe.2026.1917527

**Published:** 2026-09-14

**Authors:** Xin Li, Mingzhu Wang, Pei Du

**Affiliations:** 1 Department of Gynaecology and Obstetrics, The Affiliated Panyu Central Hospital, Guangzhou Medical University, Guangzhou, Guangdong, China; 2 Institute of Insect Science and Technology & School of Life Sciences, South China Normal University, Guangzhou, China

**Keywords:** 3D bioprinting, bioinks, decellularized extracellular matrix (dECM), organ on a chip, regenerative medicine, tissue engineering

## Abstract

Decellularized extracellular matrix (dECM) is a biomaterial generated by the selective removal of cellular components from native tissues while preserving the three-dimensional ultrastructure and endogenous biochemical cues of the extracellular matrix. Owing to its favorable biocompatibility, low immunogenicity, and capacity to mimic tissue-specific microenvironments, dECM has been increasingly explored across a wide range of biomedical applications. Its role has expanded from a conventional scaffold for tissue repair to a multifunctional biomaterial platform integrating 3D bioprinting bioinks, organ-on-a-chip matrices, organoid culture systems, and immunomodulatory applications. This review summarizes recent advances in dECM preparation, engineering applications, and clinical translation between 2020 and 2026. In terms of preparation methodology, conventional single physical, chemical, or enzymatic decellularization strategies are being replaced by integrated and tissue-specific combinatorial approaches. Emerging techniques, including supercritical CO_2_ treatment, macromolecular crowding, and multi-index-guided automated processing, provide new routes for standardized and scalable dECM production. In engineering applications, dECM has achieved key breakthroughs in 3D bioprinting, organ-on-a-chip platforms, and tissue repair, marking a paradigm shift from structural support to functional regulation. In clinical translation, dECM products have demonstrated promising therapeutic potential in hard-to-heal wound repair. Finally, this review discusses the major technical and translational challenges currently facing the dECM field, including insufficient standardization of preparation protocols, potential immunogenic risks, suboptimal mechanical matching, and incomplete regulatory frameworks. Future directions, including precision whole-organ decellularization, programmable immunomodulation, and intelligent manufacturing, are further discussed to inform the continued development and clinical translation of dECM-based biomaterials.

## 1 Introduction

Tissue engineering and regenerative medicine aim to repair, replace, or functionally reconstruct damaged tissues and organs by integrating seed cells, functional scaffolds, and bioactive factors. These strategies rely on either *in vitro* biomimetic construction or *in situ* induction of tissue regeneration and represent an important frontier in contemporary regenerative medicine. A central challenge in this field is the development of functional biomaterial platforms that can accurately recapitulate native tissue microenvironments and regulate cellular behavior in a controlled manner. dECM offers a promising solution to this challenge because of its inherent structural and biochemical similarity to native tissues. Through combined physical, chemical, and enzymatic decellularization processes, cellular antigens can be effectively removed, thereby reducing the risk of post-transplantation immune rejection, while the native three-dimensional matrix network composed of collagen, elastin, glycosaminoglycans (GAGs), and various endogenous growth factors can be largely preserved (Jin et al., 2025). Foundational studies by Badylak and colleagues have demonstrated that native ECM is not merely a structural support material, but also a bioactive signaling hub that actively regulates key biological processes, including morphogenesis, tissue homeostasis, wound repair, and regeneration. This makes ECM a natural regenerative matrix that remains difficult to replicate using synthetic materials (Badylak et al., 2009; Hussey et al., 2018).

Between 2020 and 2026, dECM research has undergone continuous expansion from feasibility-oriented preparation toward more precise, functional, and mechanism-driven applications. Early studies mainly focused on the exploration and optimization of individual decellularization methods. Perfusion-based whole-organ decellularization was first established by Ott and colleagues in 2008, who demonstrated that perfusion-based detergent treatment could generate intact cardiac extracellular matrix scaffolds while preserving native vascular architecture and tissue-specific biochemical cues, providing the foundation for subsequent whole-organ engineering strategies (Ott et al., 2008), Subsequent studies expanded this concept toward functional organ reconstruction. Petersen et al. demonstrated in 2010 that decellularized lung scaffolds could support epithelial and endothelial cell repopulation and partial functional reconstruction after implantation, representing a landmark advance from structural scaffold generation toward organ-level tissue engineering (Petersen et al., 2010), The Ott group further standardized perfusion decellularization procedures and highlighted the importance of pressure control and perfusion parameters for reproducible whole-organ scaffold fabrication (Guyette et al., 2014). In the past 5 years, progress has been made in process simplification and tissue-specific adaptation, such as the development of gravity-driven simple perfusion devices (Duarte-Ortega et al., 2025). Meanwhile, emerging approaches including supercritical fluid processing (Jeong et al., 2025) have helped address the limitations of conventional protocols, including low efficiency, poor reproducibility, and batch-to-batch variability, thereby supporting the standardized, reproducible, and scalable production of dECM. Despite these advances, Jin and colleagues noted in 2025 that several key issues continue to limit the clinical translation of dECM, including the lack of unified decellularization standards, residual matrix immunogenicity, insufficient mechanical performance, and limited structural durability (Jin et al., 2025).

At the same time, the application scope of dECM has continued to expand. dECM is moving beyond its traditional role as a passive structural scaffold and is increasingly being developed as a multifunctional platform for diverse biomedical applications. In engineered biomimetic fabrication, macromolecular crowding has enabled high-precision 3D printing of unmodified native dECM, addressing the difficulty of shaping and printing native matrices without chemical modification (Jordan et al., 2025). Photo-activated modified dECM bioinks can significantly improve printing fidelity and structural stability while preserving the native bioactivity of the matrix, thereby balancing processability with biological function (Kim M. et al., 2025). Tumor organ-on-a-chip systems constructed using dECM can closely mimic the tumor immune microenvironment and dynamically reproduce immune-cell infiltration, recognition, and killing of tumor organoids, providing useful *in vitro* models for cancer mechanism studies and high-throughput drug screening (Li Y. et al., 2026). In clinical translation, a real-world clinical study of a porcine placenta-derived dECM product (PPECM; InnovaMatrix® AC, Contavia Inc., USA) reported a 12-week wound closure rate of 62.9% in 89 patients with refractory wounds, indicating stable and clinically relevant wound-healing outcomes (Soong et al., 2025). Collectively, these advances suggest that dECM has evolved from an acellular matrix scaffold into a multifunctional natural biomaterial platform that integrates structural support, biochemical signaling, mechanical microenvironmental adaptation, and immune microenvironment modulation.

This review focuses on the evolution of dECM as a multifunctional biomaterial platform and summarizes representative advances in the field from 2020 to 2026. First, recent developments in dECM preparation technologies are reviewed, with emphasis on the advantages and limitations of different decellularization strategies. Second, the applications of dECM are discussed in three major areas: 3D bioprinting, organ-on-a-chip and organoid culture, and tissue-specific *in vivo* regenerative repair. These sections highlight the role of dECM in connecting *in vitro* biomimetic modeling with *in vivo* precision repair. Finally, key translational barriers, including process standardization, immunological safety, mechanical matching, and industrial regulation, are analyzed, and future directions such as precision customization of dECM, programmable immunomodulation, intelligent manufacturing, and clinical-scale translation are discussed.

To provide an overview of this evolutionary trajectory, we summarize the transition of dECM from a structural scaffold to a multifunctional biological platform and highlight its emerging applications and future translational opportunities (Figure 1).

**FIGURE 1:**
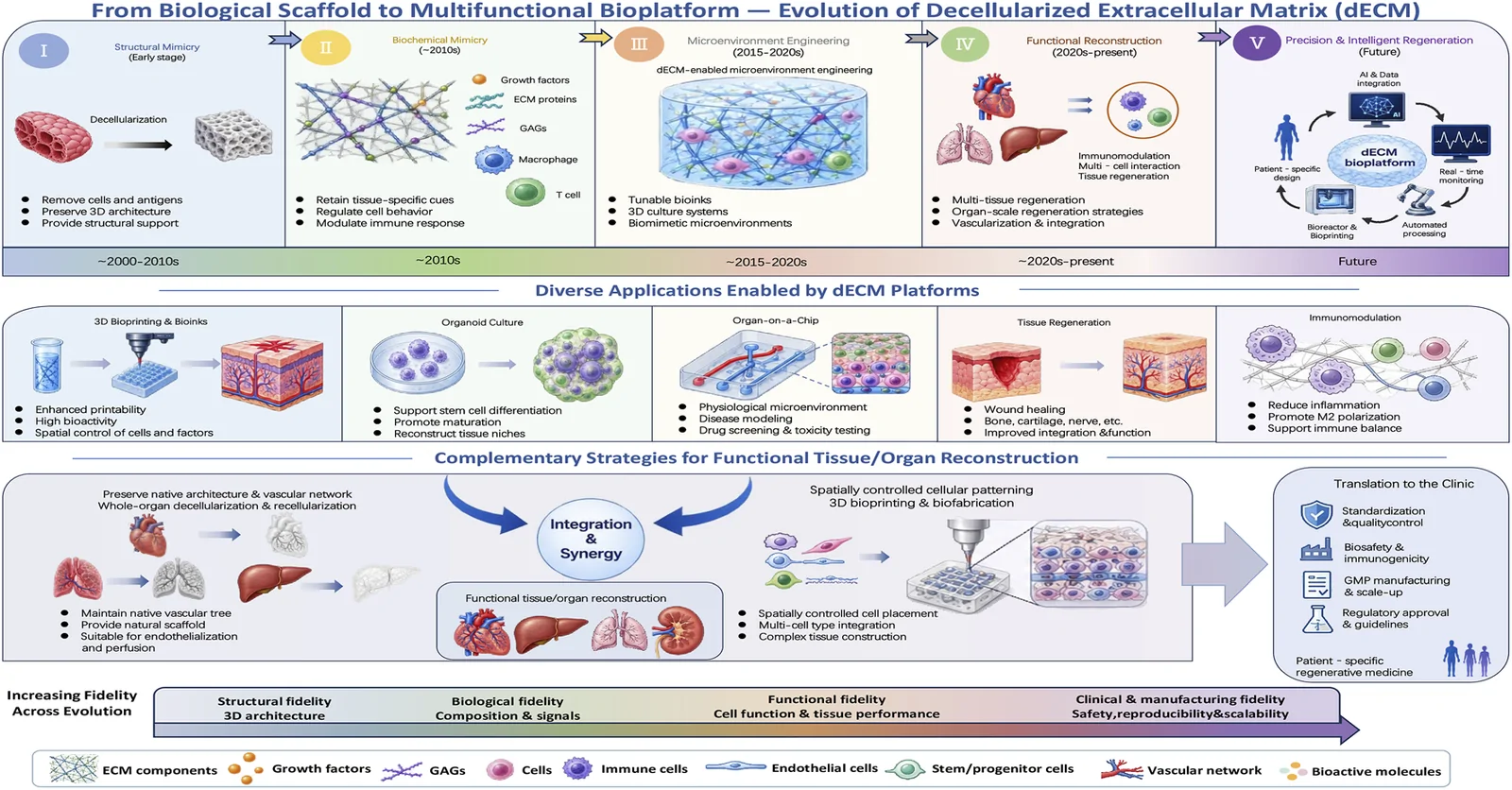
Evolution of decellularized extracellular matrix (dECM) from biological scaffold to multifunctional bioplatform.

The development of dECM technologies has progressed through several conceptual transitions, from structural mimicry and passive mechanical support toward biochemical regulation, microenvironment engineering, functional tissue reconstruction, and precision regenerative medicine. Early decellularization approaches focused on removing cellular components while preserving native three-dimensional architecture and tissue-specific extracellular matrix composition. Subsequent advances revealed that dECM retains diverse bioactive cues, including matrix proteins, glycosaminoglycans, growth factors, and immunomodulatory signals, enabling active regulation of cell behavior and tissue repair. Recent engineering strategies, including dECM bioinks, organoid culture systems, organ-on-chip platforms, and immunomodulatory biomaterials, have expanded dECM applications beyond traditional scaffolding. Complementary integration of whole-organ decellularization/recellularization and spatially controlled biofabrication provides new opportunities for functional tissue and organ reconstruction. Future development of dECM platforms will rely on precision manufacturing, intelligent monitoring, standardization, and regulatory advancement to facilitate clinical translation.

## 2 Preparation and characterization methods

Different from the “single-method exploration” paradigm before 2020, the most fundamental change in dECM preparation technologies between 2020 and 2026 lies in the transition from traditional single-treatment approaches to multi-technology synergistic intelligent systems. The central challenge in decellularization is to achieve efficient removal of cellular components while preserving the structural integrity and biological activity of the native matrix. An ideal decellularization protocol should thoroughly remove immunogenic components, including cell nuclei and residual DNA, while preserving the native three-dimensional ECM architecture, functional proteins, and growth factor activity as much as possible. As early as 2011, Crapo and colleagues pointed out that no single physical, chemical, or enzymatic method can simultaneously ensure high decellularization efficiency and adequate matrix preservation (Crapo et al., 2011). Indeed, tissue-specific multi-technique sequential combination protocols have now become the new industry standard, and intelligent process design based on multi-parameter real-time control is further advancing the development of more precise and standardized dECM production.

### 2.1 Physical methods: creating pretreatment pathways for mild decellularization

Physical decellularization methods mainly rely on mechanical action, energy input, or physical environment modulation to induce cell membrane disruption, promote cellular component release, and improve internal mass transport within tissues (Gilbert et al., 2006). Compared with traditional strategies that rely solely on chemical detergents or enzymatic treatment, physical stimulation can enhance tissue openness to a certain extent and facilitate the penetration of subsequent decellularization reagents. However, physical treatment alone often results in substantial residual nuclear material, especially in dense and complex tissues, and is therefore more suitable as a pretreatment step rather than as a standalone decellularization method. In recent years, the role of physical methods has shifted from independent decellularization strategies to pretreatment approaches that facilitate the penetration of subsequent chemical and enzymatic reagents, thereby improving the overall efficiency of decellularization while reducing chemical reagent intensity and maximizing ECM structure and bioactivity preservation (Shevchuk et al., 2025). With the development of novel physical auxiliary technologies such as ultrasound and supercritical fluids, physical modulation strategies are becoming an important technical direction for constructing high-quality, highly bioactive dECM materials.

Ultrasound-assisted decellularization is a relatively mature physical pretreatment strategy. In 2021, Lin and colleagues applied an ultrasound-assisted protocol to human umbilical arteries (HUAs). By combining 204 W ultrasound pretreatment for 4 h with 2% SDS washing for 24 h, this protocol removed more than 90% of residual tissue DNA while preserving the three-dimensional ECM architecture, mechanical compliance, and key matrix proteins, including type I/III collagen, elastin, fibronectin, and laminin. The resulting decellularized vascular scaffolds supported the adhesion and proliferation of human umbilical vein endothelial cells. After implantation into the rat abdominal aorta for 90 days, the scaffolds remained patent and gradually underwent endothelial remodeling and smooth muscle cell infiltration, indicating favorable biocompatibility. In addition, this protocol reduced the decellularization time from the conventional 28 h–16 h and decreased the amount of chemical detergent required, thereby improving both efficiency and safety (Lin et al., 2021).

Supercritical CO_2_ (scCO_2_) processing is another emerging physical approach with considerable potential. Owing to its high permeability and near-zero surface tension, scCO_2_ can penetrate dense tissues and disrupt cells *in situ*, while also providing a degree of sterilization. These properties make it particularly suitable for the decellularization of dense tissues. In 2024, Arslan and colleagues compared seven different decellularization protocols for bovine spinal cord meninges. The scCO_2_-based protocol was completed within 3 days, compared with 7 days for conventional methods, while preserving tissue structure and mechanical integrity. The resulting hydrogels also showed high cell viability and good biocompatibility (Ozudogru et al., 2024). In 2025, Salingaros and colleagues developed a scCO_2_-based injectable human-derived acellular adipose scaffold without the use of harsh chemical detergents. The final product had a dsDNA content of 44 ng/mg, which was substantially lower than that of the non-decellularized control tissue (125 ng/mg) (Salingaros et al., 2025). In the same year, Jeong and colleagues used a supercritical fluid system (scCO_2_-EtOH) to process porcine adipose and cardiac tissues. The dECM produced by this method retained adipogenic and angiogenic cytokine activity at levels 1. Five- to 5-fold higher than those achieved using conventional protocols. The authors further incorporated this highly bioactive dECM into carboxymethyl chitosan (CMC) to construct a dual-crosslinked thermosensitive hydrogel. The resulting filler material exhibited low injection resistance and favorable thermosensitive gelation properties. In a UVB-induced skin photoaging mouse model, the composite hydrogel suppressed inflammatory responses, promoted angiogenesis, upregulated matrix regeneration-related factors such as VEGF, collagen, elastin, and CTGF, and enhanced skin repair and remodeling. Its overall performance was superior to that of pure hyaluronic acid and CMC alone (Jeong et al., 2025). Collectively, these studies suggest that scCO_2_-based decellularization, with its advantages of high permeability, low residue, and simultaneous sterilization, is particularly suitable for mild and efficient processing of dense tissues and may provide a greener route for producing biosafe and bioactive dECM scaffolds and injectable hydrogels.

Tissue-specific physical pretreatment strategies have also continued to develop for challenging dense tissues. In 2025, Dortaj and colleagues focused on articular cartilage, a tissue characterized by low cellularity and high matrix density, and developed a predominantly physical decellularization protocol. Through the combined use of freeze-thaw cycling, mechanical agitation, and gradient centrifugation, this method avoided the degradation of key cartilage components, including GAGs and type II collagen, that can be caused by chemical reagents. This work provides a new strategy for mild and high-fidelity decellularization of dense soft tissues (Dortaj et al., 2024). In the same year, Mathur and colleagues developed a combined strategy using low-concentration detergents and repeated freeze-thaw cycles for esophageal tissue decellularization. This protocol preserved the aligned fiber structure of the esophageal muscular layer while reducing residual DNA to within the accepted standard, thereby balancing structural preservation with decellularization efficiency (Mathur et al., 2025).

### 2.2 Chemical and enzymatic methods: balancing decellularization efficiency and matrix integrity

Chemical detergents are currently the most widely used key components in decellularization processes, primarily achieving efficient decellularization by disrupting cell membrane structures, dissolving cellular lipids, and promoting the release of cellular components. However, different types of detergents present a clear trade-off between cell removal efficiency and ECM functional preservation, and the choice of detergent has long been a subject of debate in the decellularization field. The ionic detergent sodium dodecyl sulfate (SDS) has strong protein denaturation and cell lysis capabilities, enabling highly efficient removal of cellular residues. However, its strong protein-denaturing action may cause damage to collagen ultrastructure, loss of glycosaminoglycans (GAGs), and reduction of growth factor activity. In contrast, the non-ionic detergent Triton X-100 is more gentle on protein conformation and ECM structure, better preserving matrix bioactivity, but its cell removal capability is relatively limited, especially for high-cell-density or dense tissues where complete decellularization is often difficult to achieve. In addition, the bile salt detergent sodium deoxycholate (SDC) has strong membrane-dissolving ability and shows high efficiency in decellularization of certain tissues. The zwitterionic detergent CHAPS, which combines protein-solubilizing capacity with relatively low denaturing effects, is used in decellularization systems requiring further preservation of ECM structure. Therefore, there is currently no “optimal” detergent applicable to all tissue types, and the synergistic optimization of detergent type, concentration, treatment duration, and perfusion parameters has become a critical factor determining decellularization outcomes.

Based on the complementary mechanisms of different detergents, sequential combination strategies have gradually become an important direction for decellularization technology optimization in recent years. Early studies have demonstrated that the combined use of multiple detergents can improve cell removal efficiency while reducing ECM damage caused by single potent detergents. For example, Ott and colleagues used a combined SDS and Triton X-100 perfusion protocol to prepare whole-heart dECM scaffolds from porcine hearts, achieving effective cell removal while preserving cardiac ECM structure and mechanical properties (Guyette et al., 2014). Furthermore, while Weiss and colleagues pioneered the concept of tissue-engineered vascular constructs based on native ECM, subsequent studies have further demonstrated significant synergistic effects among different detergents. For instance, combination strategies involving Triton X-100, SDC, and SDS can balance cell lysis efficiency and ECM preservation, providing optimized pathways for complex vascular tissue engineering (Laker et al., 2020).

In recent years, research has further evolved from empirical combinations toward tissue-specific optimization. In 2025, Puthiya Veettil and colleagues used whole-organ porcine liver decellularization as a model to systematically compare the effects of SDS alone, Triton X-100 alone, and sequential SDS/Triton X-100 treatment on dECM quality. The results showed that the sequential protocol, in which SDS was first used for efficient removal of cellular components and Triton X-100 was subsequently applied for gentler preservation of matrix structure, was superior to either single-detergent protocol in terms of DNA removal, GAG retention, and preservation of key growth factors such as HGF and VEGF. This approach therefore provides an optimized protocol framework for the standardized preparation of whole-organ liver dECM scaffolds (Puthiya Veettil et al., 2025). Similarly, Pfarr and colleagues systematically compared the effects of trypsin, SDS, and Triton X-100 on decellularization efficiency and functional performance of porcine vascular grafts in 2025. They found that although SDS and trypsin achieved higher degrees of cell removal, they also caused partial ultrastructural damage. In contrast, vascular matrices treated with Triton X-100 exhibited better cell adhesion and endothelialization capacity, suggesting that decellularization efficiency alone cannot represent material quality, and that cell repopulation capacity and functional recovery should also be incorporated into the evaluation system (Pfarr et al., 2025).

Overall, the development trend of chemical decellularization strategies in recent years has shifted from simply pursuing “maximal cell removal” toward “precisely balancing decellularization efficiency and ECM functional preservation. “ Future research needs to integrate the structural characteristics of different tissues to achieve more standardized and tissue-specific decellularization processes through optimization of detergent combinations, concentration gradients, perfusion modes, and real-time quality monitoring systems.

However, relying solely on detergent systems still cannot fully address issues such as insufficient clearance of residual nucleic acids and the difficulty of disrupting specific cell-matrix connections during decellularization. Therefore, enzymatic decellularization typically serves as an important complement to chemical treatment, utilizing the specific degradation capabilities of enzymes to achieve removal of residual cellular components and regulation of tissue structure. In recent years, the focus of enzymatic strategies has also gradually shifted from traditional broad-spectrum degradation modes toward more precise dose control, temporal regulation, and process monitoring, to further improve decellularization efficiency and minimize ECM functional loss.

Enzymatic decellularization is undergoing a paradigm shift from “broad-spectrum and crude” toward “precise dosing and process control,” with developments primarily centered around two technical trajectories: optimization of nucleic acid removal systems and precise regulation of proteolytic processes. First, nuclease systems are gradually becoming more standardized and simplified. DNase and RNase have become standard ancillary treatments for residual nucleic acid removal after decellularization, and the widely adopted quality evaluation guidelines typically recommend that residual double-stranded DNA content in dECM be below 50 ng/mg dry weight, with fragment lengths below 200 bp, to minimize immunogenic risks (Wang et al., 2026). In 2024, Ramos and colleagues compared the nucleic acid removal efficacy of a Benzonase single-enzyme system with a Pulmozyme plus RNase T1 dual-enzyme system in human dermis decellularization. They found that Benzonase treatment achieved a residual DNA content of 9. 97 ng/mg (99. 8% removal), which was not significantly different from the dual-enzyme system (99. 9%), while offering advantages in storage stability and cost control. This suggests that simplified nuclease combinations may become an important direction for future standardized decellularization workflows (Stern et al., 2025). Second, the application of proteolytic enzymes is gradually shifting from non-specific degradation toward precisely controlled spatiotemporal regulation. Trypsin, as a representative proteolytic enzyme, primarily facilitates cell dissociation by cleaving cell-matrix adhesion connections (such as integrin-mediated interactions and fibronectin networks). However, its non-specific cleavage activity poses a potential threat to collagen fibers—excessive concentration or prolonged treatment can lead to significant degradation of matrix mechanical properties. Therefore, current research tends to position trypsin as an auxiliary decellularization tool, using low concentrations and short treatment times to achieve cell dissociation while avoiding ECM structural damage that could result from its use as a primary decellularization agent (Pfarr et al., 2025).

Overall, the development trend of enzymatic strategies has shifted from simply optimizing “which enzyme to use” toward regulating “when, where, and to what extent the enzyme acts. “ However, dECM from different tissue sources has different requirements for enzymatic treatment parameters due to variations in cell density, ECM crosslinking degree, and tissue structure. Traditional methods relying on empirical determination of reaction endpoints struggle to ensure batch-to-batch consistency. Therefore, recent research has begun exploring intelligent decellularization processes based on real-time monitoring and feedback control. In 2025, Kim and colleagues proposed a multi-index-dependent decellularization process that monitors key parameters in the eluate in real time, including DNA concentration, total protein concentration, and turbidity, and dynamically adjusts detergent concentration and perfusion flow rate. This approach enabled scalable and standardized preparation of tissue-specific dECM bioinks (Kim H. J. et al., 2025). Such process-control strategies represent an important step from experience-based manual operation toward quantitative and engineering-oriented dECM manufacturing.

### 2.3 Combinatorial strategies and standardization: from tissue-specific adaptation to intelligent process design

Standardization of decellularization protocols and quality-control systems is essential for translating dECM from laboratory research to large-scale clinical application. A broad consensus has emerged that there is no universal decellularization protocol suitable for all tissues. Instead, protocol design and parameter optimization must be adapted to tissue-specific characteristics, including cellular density, matrix composition, and structural compactness. In 2026, a systematic review published in Biomedical Technology summarized optimized decellularization parameter combinations for different tissue types, including skin, bone, nerve, heart, lung, liver, and kidney. It also proposed a three-dimensional evaluation framework incorporating DNA removal efficiency, ECM structural integrity, and bioactivity retention, thereby providing a reference framework for standardized assessment of decellularization outcomes (Wang et al., 2026). From the perspective of clinical translation, Laude and colleagues further emphasized in 2025 that the central challenge in the field is not simply the development of new decellularization techniques, but the establishment of unified, tissue-specific characterization standards and batch-release quality-control systems that are comparable across laboratories and feasible for industrial implementation. This is critical for addressing the persistent issue of batch-to-batch variability in dECM products (Laude et al., 2026).

In parallel, simplified and cost-effective decellularization platforms are also being developed to improve process accessibility. A study published in 2025 reported a gravity-driven perfusion decellularization device that enabled stable whole-liver decellularization in rats without the need for expensive peristaltic pumps or precise pressure-control systems. The resulting liver dECM scaffold retained the native vascular network and exhibited favorable hepatocyte compatibility and biological activity. Because of its simplicity and low cost, this approach provides an accessible technical option for dECM research in resource-limited laboratories (Duarte-Ortega et al., 2025).

### 2.4 Comparative evaluation and future perspectives of decellularization strategies

Overall, physical, chemical, enzymatic, and combinatorial decellularization strategies each have distinct characteristics in terms of cell removal efficiency, ECM structural integrity, bioactivity retention, processing time, and applicable tissue types. However, there is currently no “universally optimal method” that can simultaneously achieve high decellularization efficiency, low immunogenicity, and maximal preservation of tissue-specific functions. Physical strategies generally offer advantages such as minimal ECM damage and low chemical residue risk, but often fail to achieve complete decellularization when used alone, and are therefore more suitable as pretreatment or adjunctive enhancement measures. Chemical strategies have high cell removal efficiency, but ionic detergents may cause collagen structural damage and loss of GAGs and active factors, while non-ionic detergents suffer from insufficient decellularization efficiency. Enzymatic strategies can specifically remove cell adhesion components and nucleic acid residues, but their action requires strict control to avoid excessive ECM degradation. Therefore, the current trend in dECM preparation strategies has gradually shifted from single-method optimization toward tissue feature-based combinatorial decellularization design, achieving a dynamic balance between decellularization efficiency and ECM functional preservation through the synergistic action of different technical approaches. Establishing tissue-specific decellularization workflows based on different tissue characteristics, combined with multi-index real-time monitoring and intelligent process control, is expected to further improve the stability, reproducibility, and scalability of dECM production.

To systematically compare the technical characteristics, applicable scopes, and key advantages and limitations of different decellularization strategies, this review summarizes representative physical, chemical, enzymatic, and combinatorial decellularization methods (Table 1).

**TABLE 1:** Comparative evaluation of current decellularization strategies for dECM fabrication.

Method category	Specific technique/Reagent	Mechanism of action	Advantages	Limitations	Representative tissues	Typical applications	References
Physical	Repeated freeze-thaw cycling	Ice crystal formation disrupts cell membranes, releasing cellular contents; often used as pretreatment	Simple operation, no chemical residues, minimal damage to matrix proteins	Low decellularization efficiency when used alone, substantial nuclear residue, requires combination with other methods	Esophagus, cartilage	Pretreatment for dense tissues to enhance subsequent chemical reagent penetration	(Mathur et al., 2025) (Dortaj et al., 2024)
	Ultrasound-assisted	Cavitation effects generate micro-jets and shock waves that mechanically rupture cell membranes	Shortens processing time (e.g., from 28 h to 16 h), reduces detergent usage, preserves collagen and elastin	Parameters (power, time) require fine optimization; excessive ultrasound may damage fiber structure	Human umbilical artery	Preparation of small-caliber vascular decellularized scaffolds for vascular tissue engineering	(Lin et al., 2021)
	Supercritical CO_2_ (scCO_2_)	High permeability of supercritical fluid penetrates tissues, dissolves cell membrane lipids, with simultaneous sterilization	Short processing time (3 days vs. 7 days conventional), no toxic chemical residues, preserves growth factor activity (1. 5–5× conventional)	High equipment cost, strict requirements for operating conditions (pressure, temperature)	Bovine spinal cord meninges, porcine adipose, cardiac	Preparation of highly active injectable hydrogels for skin photoaging repair and soft tissue filling	(Ozudogru et al., 2024) (Jeong et al., 2025) (Salingaros et al., 2025)
Chemical	Ionic detergent (SDS)	Disrupts cell membrane phospholipid bilayers, solubilizes nuclear proteins, efficiently removes cellular components	Extremely high decellularization efficiency, DNA residues can be below 50 ng/mg	Excessive use damages ECM ultrastructure, reduces GAG and growth factor activity, affects biomechanical properties	Liver, blood vessels	Whole-organ perfusion decellularization (liver), preparation of large-scale scaffolds	(Puthiya Veettil et al., 2025) (Pfarr et al., 2025)
	Non-ionic detergent (Triton X-100)	Gently lyses cell membranes, preserves protein conformation and matrix ultrastructure	Minimal damage to ECM structure, good bioactivity preservation	Relatively low decellularization efficiency, often leaves substantial DNA residues when used alone, requires extended processing time	Blood vessels, skin	Decellularization of soft tissues requiring preservation of delicate collagen structure	(Pfarr et al., 2025)
Enzymatic	Trypsin	Cleaves cell-matrix adhesion proteins (e.g., integrins, fibronectin), promoting cell detachment	Effectively removes cellular debris from ECM, rapid action	Non-specific protease; excessive concentration or prolonged treatment degrades collagen and elastin, weakening mechanical properties	Blood vessels, skin	Typically combined with detergents to enhance cell detachment	(Pfarr et al., 2025)
	Nucleases (DNase/RNase, Benzonase)	Degrades residual DNA/RNA, reducing immunogenicity	Efficiently removes nucleic acid residues (Benzonase up to 99. 8% removal), lower cost than enzyme combinations	Acts only on nucleic acids, cannot achieve decellularization alone, requires combination with other methods	Human dermis	Post-decellularization treatment to ensure DNA <50 ng/mg standard	(Stern et al., 2025)
Combinatorial	Sequential SDS → Triton X-100 perfusion	SDS first efficiently removes cells, Triton then gently washes, synergistically preserving matrix components	Balances high decellularization efficiency with GAG and HGF/VEGF preservation, superior to single agents	Longer protocol, requires optimization of switching timing and concentration	Porcine whole liver	Preparation of bioactivity-preserving whole-liver decellularized scaffolds for liver regeneration	(Puthiya Veettil et al., 2025)
	Freeze-thaw + low-concentration detergent	Physical freeze-thaw pre-disrupts cell membranes, then low-concentration SDS or Triton clears residues	Reduces chemical reagent usage, preserves matrix architecture (e.g., esophageal muscle layer aligned fibers)	Freeze-thaw parameters (cycle number, rate) require tissue-specific optimization	Rat esophagus	Preparation of esophageal decellularized matrix for esophageal repair scaffolds	(Mathur et al., 2025)
	Ultrasound + SDS	Ultrasound pretreatment increases reagent penetration, SDS efficiently decellularizes	Significantly shortens total time (16 h vs. 28 h), reduces SDS usage, maintains mechanical compliance	Ultrasound power requires precise control to avoid localized overheating damage	Human umbilical artery	Rapid preparation of small-caliber vascular scaffolds that remain patent and undergo remodeling after implantation	(Lin et al., 2021)
	Multi-index-dependent automated process	Real-time monitoring of eluate DNA, total protein, turbidity to dynamically adjust detergent concentration and flow rate	Achieves batch-to-batch consistency, suitable for scalable dECM bioink production	Requires online detection system, high initial equipment investment	Multiple tissues (heart, liver, *etc.*,)	Standardized production of tissue-specific dECM bioinks for 3D bioprinting	(Kim et al., 2025a)

As shown in Table 1, there is currently no single protocol that can be applied to all tissue sources while simultaneously achieving high decellularization efficiency, ECM integrity preservation, and bioactivity retention. Different decellularization strategies inherently involve trade-offs between cell removal efficiency and matrix functional preservation. Therefore, the future focus of dECM preparation technology development is not to find a single optimal method, but to establish personalized decellularization systems based on tissue characteristics, application requirements, and target functions. Combinatorial decellularization strategies and multi-parameter feedback-based intelligent process control technologies will become important directions for improving decellularization efficiency, reducing batch-to-batch variation, and achieving standardized production, providing critical support for the translation of dECM from laboratory preparation to scalable manufacturing and clinical application.

### 2.5 Advanced characterization technologies for dECM: from component identification to functional profiling

Traditional histological staining, biochemical quantification, and DNA residue detection are primarily used to evaluate the degree of decellularization and basic composition of dECM. However, these methods are insufficient to reveal the complex molecular differences among dECM from different tissue sources, nor can they explain the functional heterogeneity in immunomodulation, cell fate determination, and tissue regeneration processes. In recent years, with the development of high-resolution mass spectrometry, multi-omics analysis, and database annotation systems, dECM characterization strategies have gradually shifted from traditional “component detection” models toward “functional profiling” models, enabling researchers to understand the relationship between ECM composition and biological activity at the molecular level.

The development of proteomics technology represents a significant breakthrough in advancing precise dECM characterization. High-throughput analytical methods based on liquid chromatography-tandem mass spectrometry (LC-MS/MS) can simultaneously identify large numbers of ECM-related proteins, not only assessing the preservation of major structural proteins such as collagen and elastin, but also resolving changes in basement membrane proteins, proteoglycans, matricellular proteins, and various cell signaling-related molecules, thereby establishing source-specific dECM molecular composition profiles. Compared with traditional biochemical assays, proteomics further reveals that dECM is not simply a structural scaffold, but a complex biological information repository containing numerous potential regulatory signals. Naba and colleagues, through large-scale proteomic analysis, proposed the “matrisome” concept, systematically classifying core ECM components (core matrisome) and matrisome-associated proteins, providing an important framework for understanding the molecular heterogeneity of ECM from different tissues. Their study demonstrated that ECM from different tissue sources has highly specific protein composition characteristics, suggesting that tissue origin itself may be a critical parameter determining dECM biological function, rather than merely representing a material source difference (Naba et al., 2012). In recent years, Anna-Maria and colleagues used LC-MS/MS technology to systematically analyze the proteomes of mouse liver- and kidney-derived dECM, revealing significant differences in collagen subtypes, basement membrane proteins, and proteoglycan composition between dECM from different organs, further indicating that tissue origin is an important molecular basis determining dECM biological function (Diedrich et al., 2024). Similarly, for tissue-specific biomaterial design, Mika and colleagues compared the protein composition and cellular responses of tissue-specific dECM hydrogels, finding that origin-specific ECM protein profiles can influence cell adhesion, migration, and tissue remodeling behavior, providing a theoretical basis for developing tissue-matched regenerative materials (Brown et al., 2023).

Beyond tissue origin analysis, proteomics is also increasingly being used to discover disease-associated ECM features. For example, in pancreatic ductal adenocarcinoma research, Considine and colleagues established tumor-derived ECM molecular signature profiles through in-depth proteomic analysis and identified ECM signatures correlated with lymphocyte infiltration and patient prognosis, providing new research avenues for using dECM to model disease microenvironments and identify potential therapeutic targets (Considine et al., 2026). However, relying solely on protein composition analysis is still insufficient to fully explain the complex biological effects of dECM. ECM function depends not only on protein types but is also regulated by protein spatial structure, post-translational modifications, crosslinking degree, and glycosaminoglycan (GAG) composition. As early as 2016, Saldin and colleagues demonstrated that decellularization processes may cause partial GAG loss, thereby reducing ECM’s ability to bind and store growth factors and further affecting its capacity to regulate cellular behavior (Saldin et al., 2017). Therefore, future dECM evaluation systems need to expand from simple protein composition analysis to multidimensional evaluation models integrating structural omics, glycoproteomics, and functional validation.

Meanwhile, ECM research is evolving from individual protein identification toward systematic database integration. In recent years, multi-omics databases and functional annotation systems have been continuously improving, enabling standardized comparison and functional prediction of ECM data from different tissue sources. With the rapid accumulation of ECM multi-omics data, standardized databases and functional annotation systems are gradually becoming important foundations for promoting dECM functional analysis. The Gene Ontology Consortium has been continuously updating the functional annotation framework for ECM-related proteins in recent years, providing data support for integrating ECM proteomics data from different tissue sources, conducting functional enrichment analysis, and building cross-study comparison systems (Aleksander et al., 2026). The molecular features of dECM revealed by the above multi-omics characterization technologies constitute the material basis for its functional regulatory roles in applications such as 3D bioprinting, organ-on-a-chip, and tissue repair.

## 3 Application frontiers: research progress in multifunctional dECM bioplatforms

Before 2020, dECM application research largely focused on the singular question of “whether it can serve as a scaffold to support cell growth. “ Research in the past 5 years has transcended this boundary, redefining dECM as “an informational biomaterial capable of actively programming cell fate, coordinating multicellular behaviors, and responding to dynamic microenvironmental changes. “ This conceptual advancement has directly driven the paradigm shift of dECM from a passive scaffold to a multifunctional bioplatform. While preparation methods define the baseline quality of dECM materials, recent application-oriented studies have further expanded the functional role of dECM beyond that of a conventional structural scaffold. dECM is increasingly being used as a multifunctional and bioactive platform that supports tissue-specific modeling, functional biofabrication, and endogenous repair regulation. This section reviews recent advances in dECM applications from three perspectives: 3D bioprinting, organ-on-a-chip and organoid culture, and tissue-specific repair.

### 3.1 dECM bioinks and 3D bioprinting: from structural mimicry to functional reconstruction

The conversion of dECM into printable bioinks represents an important development direction in tissue engineering and biofabrication in recent years. Unlike traditional synthetic polymers or single natural polymer materials, the greatest advantage of dECM bioinks lies in their retention of source tissue-specific ECM composition and bioactive signals, enabling printed three-dimensional structures not only to provide spatial support but also to actively regulate cell adhesion, migration, differentiation, and tissue remodeling processes. Therefore, the development of dECM bioprinting has evolved from the early “proof-of-concept” stage of demonstrating that native ECM is printable, toward the precision manufacturing stage that balances printing resolution, mechanical properties, and tissue functional reconstruction.

The early breakthrough in dECM bioprinting came from the pioneering work of the Dong-Woo Cho team. In 2014, Pati and colleagues first reported a three-dimensional bioprinting strategy based on tissue-derived dECM. By preparing cardiac-derived dECM as a printable hydrogel and combining it with cell loading to construct three-dimensional structures with tissue-specific microenvironments, they demonstrated that native ECM can serve not only as a traditional tissue engineering scaffold material but also as a bioactive printing ink for complex tissue fabrication (Pati et al., 2014). This study established the foundation for dECM as a functional bioink and promoted the establishment of printing systems for ECM from different tissue sources. However, early dECM printing systems were still limited by the inherent properties of native ECM hydrogels, including low viscosity, slow gelation, and insufficient mechanical support, leading to easy collapse of printed structures and limiting their application in complex, large-scale tissue construction.

In the past 5 years, important advances in dECM bioprinting have primarily focused on addressing the aforementioned “insufficient printability” technical bottleneck. In 2022, the Feinberg team applied FRESH (Freeform Reversible Embedding of Suspended Hydrogels) 3D bioprinting technology to dECM systems, developing patient-specific dECM printed scaffolds for volumetric muscle loss (VML) repair. Through CT image reconstruction combined with FRESH printing, this study achieved the fabrication of large-size, high-fidelity dECM scaffolds and validated their therapeutic potential in animal VML models, providing important impetus for the development of dECM from traditional amorphous scaffolds toward personalized, functional tissue fabrication platforms (Behre et al., 2022).

Beyond printing method optimization, recent research has further focused on establishing optimal printing parameter windows for dECM materials themselves. In 2025, Palierse and colleagues systematically investigated the effects of different concentrations of skin-derived dECM ink on printing performance and cellular behavior. They found that although higher concentration (3% w/v) improved material modulus and structural retention, excessive stiffness restricted cell spreading and proliferation. In contrast, the 2% w/v dECM system achieved a better balance between printing stability and cell activity, maintaining extruded structural integrity while supporting fibroblasts to maintain normal morphology and proliferation capacity (Palierse et al., 2025). This study suggests that the optimization goal of dECM printing systems is not simply to increase material strength, but to establish a dynamic balance between printing performance and biological activity based on tissue requirements.

Meanwhile, materials engineering strategies are further advancing dECM printing from parameter optimization toward active regulation. Photo-crosslinking modification methods such as methacrylation can endow dECM materials with rapid light-responsive crosslinking capability, thereby improving post-printing structural stability and mechanical properties. In recent years, multiple studies have shown that photo-crosslinkable dECM systems can effectively address the issues of slow gelation and insufficient mechanical strength of native dECM, and have been applied in cartilage, skin, and vascular tissue engineering constructs (Kim M. et al., 2025; Kafili et al., 2026). However, this strategy also has potential limitations, such as residual exogenous chemical groups and photoinitiators that may affect long-term immune safety, thus requiring further evaluation of their *in vivo* application risks.

Compared with strategies relying on chemical modification, macromolecular crowding (MMC) technology represents a more biomimetic direction in dECM printing development in recent years. In 2025, Jordan and colleagues reported a printing strategy based on native ECM self-assembly regulation. By adding inert macromolecules such as polyvinylpyrrolidone (PVP) to dECM solutions, they utilized the excluded volume effect to increase the local concentration of ECM molecules, accelerating collagen fiber formation and achieving rapid gelation and three-dimensional structural maintenance of dECM solutions from multiple tissue sources (Jordan et al., 2025). Compared with traditional chemical crosslinking methods, this approach does not require the introduction of additional crosslinking groups, maintaining native ECM composition and bioactivity while improving material formability, providing new ideas for constructing dECM printing systems with high biosafety.

Overall, the development of dECM bioprinting technology shows an evolutionary trend from “material printability” toward “functional designability. “ The early work of the Cho team demonstrated the feasibility of tissue-derived dECM as a printing ink, while research in the past 5 years has primarily focused on resolving the conflicts among printing resolution, structural stability, and functional preservation. FRESH printing strategies have improved complex structure fabrication capability through manufacturing process optimization; concentration regulation strategies have established balance windows between printing performance and cellular responses; photo-crosslinking strategies have enhanced material mechanical properties through engineering modification; and MMC strategies have further explored the use of ECM’s intrinsic molecular assembly rules to achieve the unification of native property preservation and printability enhancement. In the future, the development focus of dECM bioprinting will shift from simply improving printing resolution toward tissue-specific design, intelligent manufacturing, and functional regenerative system construction.

### 3.2 Organ-on-a-chip and organoid culture: dECM for tissue-specific biomimetic microenvironment construction

The goal of 3D bioprinting is often to fabricate functional tissue constructs *in vitro*, whereas organ-on-a-chip and organoid culture systems aim to reproduce tissue function and disease processes under controlled conditions. These applications impose specific requirements on matrix materials: beyond providing physical support, the matrix must reproduce tissue-specific microenvironmental cues and induce cellular behaviors that resemble those observed *in vivo*. Conventional PDMS chips lack bioactive interfaces, and Matrigel, although widely used for organoid culture, is derived from mouse sarcoma cells and has limitations related to undefined composition, batch variability, and clinical safety. dECM, with its tissue-specific proteomic profile, growth-factor composition, and matched mechanical properties, is therefore a promising alternative to conventional culture matrices.

A 2026 study published in Talanta developed a colorectal cancer organ-on-a-chip platform based on a dECM-GelMA composite hydrogel. The platform used a 5% w/v composite hydrogel with 50–150 μm pores and a compressive modulus matched to physiological parameters of colorectal cancer, enabling long-term three-dimensional culture of patient-derived tumor organoids. Through microfluidic perfusion, the system dynamically simulated the tumor immune microenvironment and allowed real-time tracking of NK-cell-derived exosome-mediated killing of tumor organoids. The study revealed a phenomenon that could not be observed in two-dimensional culture: within the three-dimensional dECM biomimetic microenvironment, NK-cell exosomes selectively targeted the hypoxic core of tumor organoids rather than producing the non-specific killing pattern observed in conventional two-dimensional systems (Li Y. et al., 2026). This finding suggests that a native-like three-dimensional dECM microenvironment is important for recapitulating dynamic tumor-immune interactions.

In cartilage organ-on-a-chip research, the dense structure and low cellularity of native cartilage ECM make biomimetic modeling particularly challenging. In 2024, Upadhyay and colleagues developed an alginate-chitosan-dECM triple-component composite bioink to address this limitation. Under cyclic fluid shear stimulation, type II collagen and aggrecan expression in chondrocytes cultured within the composite system increased by 2. 3-fold and 1. 8-fold, respectively, compared with the pure polymer matrix group, indicating improved recapitulation of the articular cartilage microenvironment (Upadhyay et al., 2024).

Brain organoid chips represent a rapidly developing area in neural biomimetic modeling and neuroregeneration. These systems provide engineered platforms for simulating brain-specific microenvironmental cues, recapitulating aspects of neurodevelopment, and investigating mechanisms of nervous system injury and repair. In 2021, Cho and colleagues combined human brain decellularized matrix (BEM) with a microfluidic chip platform to construct a brain organoid culture system with native neural microenvironmental features. The combination of dynamic microfluidic perfusion and BEM-derived biochemical signals promoted neurogenesis, accelerated cortical layer development, increased organoid size, and enhanced electrophysiological function, while reducing batch-to-batch variability and improving model reproducibility (Cho et al., 2021). In 2024, Wu and colleagues prepared rat brain-derived decellularized ECM hydrogel (DBECMH) and applied it to the culture of human iPSC-derived spinal cord organoids. Compared with Matrigel, DBECMH increased the expression of multiple spinal-cord region-specific markers and promoted structural development and functional maturation of spinal cord organoids, indicating the potential of brain-derived dECM as an alternative matrix for reproducible neural organoid culture (Wu et al., 2024). In a parallel effort leveraging dECM in a different format, Gong and colleagues (2026) processed human lung dECM into microparticles (dECM-MPs) and co-assembled them with human bronchial epithelial cells to generate apical-out airway organoids (dECM-AoAO) (Gong et al., 2026). In this system, the epithelium directly interfaces with dECM-MPs at its basal side while maintaining an outward-facing apical surface, closely recapitulating the native apicobasal polarity of the airway epithelium. Incorporation of dECM-MPs markedly diversified the epithelial lineage composition—unlike ECM-free AoAOs, which are dominated by multiciliated cells, dECM-AoAOs readily gave rise to SCGB1A1^+^ club cells, MUC5AC^+^ goblet cells, and TP63^+^ basal stem cells, more closely mirroring the native epithelial landscape. Upon IL-13 stimulation, dECM-AoAOs exhibited canonical goblet cell hyperplasia and reduced surface ciliation, whereas ECM-free AoAOs showed minimal response to the same challenge, underscoring that basolateral matrix cues provided by dECM are critical for endowing airway organoids with pathophysiological relevance. Furthermore, the outward-facing motile cilia drove quantifiable two-dimensional organoid locomotion, which was markedly suppressed by the dynein inhibitor EHNA, offering a label-free, high-throughput functional readout of ciliary activity and tissue-level maturity. For neural injury repair and 3D biomimetic scaffold fabrication, a team from Hebei Medical University extracted dECM components from porcine brain tissue in 2025 and combined them with gelatin and sodium alginate to prepare a composite bioink. Using 3D bioprinting, the team constructed a three-dimensional neural scaffold loaded with human umbilical cord mesenchymal stem cells (hUCMSCs). The scaffold had a storage modulus of approximately 800 Pa, closely matching the mechanical properties of native brain tissue. In a rat intracerebral hemorrhage model, the scaffold reduced hematoma volume by 51. 2%, upregulated VEGF expression by 3. 1-fold, and improved neurological deficits, suggesting a potential strategy for neural repair after intracerebral hemorrhage (Zhang A. et al., 2025). In 2026, Jiang and colleagues further integrated porcine brain-derived dECM hydrogel with flexible liquid-metal multielectrode arrays to construct an electronic brain biochip. By layer-by-layer stacking of dECM hydrogels, the authors created a three-dimensional multilayer neural network and embedded flexible, rollable electrodes between matrix layers. This platform enabled dynamic, real-time monitoring of spontaneous electrical activity and chemically induced responses in three-dimensional neural networks and was validated in both rat primary neurons and hiPSC-derived neurons (Liu et al., 2026a).

Furthermore, a cell-derived pure ECM hydrogel, termed CEOgel, was first reported in 2026 and has expanded the range of matrices available for organoid culture. CEOgel is produced by inducing fibroblasts *in vitro* to secrete and self-assemble ECM, followed by decellularization and thermoreversible gelation. This strategy avoids reliance on animal tissue sources and offers advantages such as more defined composition and low immunogenicity, indicating potential for scalable organoid culture and safe cell-delivery applications (An et al., 2026).

Taken together, dECM can compensate for several limitations of conventional synthetic materials and Matrigel by providing tissue-specific biochemical components, appropriate mechanical properties, and bioactive cues. In tumor, cartilage, and neural models, dECM derived from different tissue sources can recapitulate native microenvironmental features, support organoid maturation and functional differentiation, and enable more physiologically relevant modeling of tumor-immune interactions, cartilage-specific matrix secretion, neural development, and electrophysiological activity. Integration of dECM with microfluidics, 3D bioprinting, and flexible bioelectronic devices is moving organ-on-a-chip systems from static structural mimicry toward dynamic functional monitoring and in vitro-in vivo functional integration. The emergence of cell-derived CEOgel further addresses the limitations of complex composition and restricted availability associated with animal tissue-derived dECM, opening new directions for standardized and scalable organoid modeling and clinical translation.

### 3.3 Tissue-specific repair: dECM-mediated active biological repair regulation

dECM has been applied to *in vivo* repair of multiple organs and tissues, including skin, cartilage, myocardium, liver, and blood vessels. Studies over the past 5 years indicate that dECM is not merely a passive filler scaffold, but can function as an active biological repair platform. Through the release of bioactive matrix-derived peptides, modulation of immune-cell polarization, and activation of endogenous growth-factor pathways, dECM can initiate multicellular and multi-pathway repair mechanisms, supporting a transition from passive structural filling to active regulation of tissue repair.

As dECM applications in tissue repair continue to expand, the material formulations have also gradually evolved from traditional bulk hydrogels toward granular and porous forms. Different formulations not only affect injectability and tissue adaptability, but also directly determine key biological behaviors such as cell infiltration, angiogenesis, and immunomodulation. Therefore, before discussing different tissue repair strategies, it is necessary to distinguish between the two most widely applied dECM hydrogel systems. From the perspective of formulation evolution, dECM hydrogels currently include two major systems: traditional bulk hydrogels and granular hydrogels. The former forms continuous three-dimensional networks through physical or chemical crosslinking, with porosity primarily derived from molecular-scale networks (typically 1–10 nm), and cell migration relies on matrix metalloproteinase (MMP)-mediated matrix degradation. The latter is assembled bottom-up from micron-sized dECM microgels (typically 40–90 μm), with inherent porosity derived from inter-particle packing gaps rather than the material’s own network structure. When inter-particle pore sizes exceed 54 μm, typical macroporous hydrogels can form, significantly promoting cell infiltration, angiogenesis, and tissue remodeling. The essential differences between the two systems mainly lie in three aspects: (1) structural units differ—bulk hydrogels are continuous networks while granular hydrogels consist of discrete microgel particles; (2) pore formation mechanisms differ—the former relies on crosslinking density to regulate pore size while the latter relies on particle packing to form micron-sized pores; (3) cell-material interactions differ—in bulk hydrogels, cells must actively degrade the matrix before migrating, while granular hydrogels provide natural migration channels for cells, enabling rapid infiltration and tissue remodeling. Based on inter-particle pore size, granular hydrogels can be further classified into small-pore (1–22 μm), medium-pore (22–54 μm), and large-pore (>54 μm) categories, with different pore sizes corresponding to different immune cell recruitment and tissue repair patterns (Feng et al., 2025). In recent years, macroporous granular dECM hydrogels have gradually become important development directions in skin wound repair, myocardial regeneration, and cartilage repair due to their excellent injectability, cell infiltration capability, and tissue adaptability.

Myocardial repair is one of the most active areas of dECM translational research. A 2025 Nature Communications study performed a spatiotemporal analysis of the repair mechanisms of injectable myocardial dECM hydrogel after myocardial infarction. In a rat model of acute myocardial infarction, injection of myocardial dECM hydrogel during the subacute phase preserved resident cardiac CD68^+^ macrophages and simultaneously activated several endogenous repair pathways, including fibroblast activation, lymphangiogenesis, angiogenesis, smooth muscle proliferation, and cardiomyocyte regeneration. This established a therapeutic model in which a single biomaterial intervention induced multicellular synergistic repair (Mesfin et al., 2025). These broad repair effects illustrate the platform-like function of dECM, as the diverse signaling molecules retained in the matrix act as a native signaling reservoir for regulating tissue repair.

Cutaneous wound repair is among the most clinically advanced applications of dECM. In 2025, Soong and colleagues conducted a real-world retrospective study of a porcine placenta-derived dECM product (InnovaMatrix® AC) in 89 patients with hard-to-heal wounds refractory to conventional treatment. The median wound duration at enrollment was 11. 4 weeks. After treatment with the dECM product, median wound area reduction at week four was 43. 33%, the 12-week wound closure rate was 62%, and the median time to closure was 66 days, demonstrating clinically relevant wound-healing efficacy (Soong et al., 2025). Mechanistic studies have suggested that small leucine-rich proteoglycans in dECM, such as decorin and biglycan, can bind Toll-like receptors on macrophages and promote M2 anti-inflammatory and pro-repair polarization. Bioactive peptides released during dECM degradation may also promote angiogenesis, thereby accelerating wound repair through both anti-inflammatory and pro-regenerative mechanisms (Zhang K. et al., 2025). In formulation optimization, Zhang and colleagues developed granular dECM microgels in 2025 that could be adapted to irregular wound defects. In a rat full-thickness skin defect model, granular microgels achieved a wound closure rate 1. 5-fold higher than that of conventional bulk hydrogels (Zhang K. et al., 2025). In 2026, Noshin and colleagues developed a superabsorbent decellularized human amniotic membrane microgel combining prolonged moisture retention with broad-spectrum antimicrobial activity, maintaining a stable moist microenvironment for 7 days during chronic wound healing (Noshin et al., 2026). A 2026 study by Liu and colleagues also showed that acellular dermal matrix hydrogel promoted re-epithelialization and ordered collagen remodeling in cutaneous wounds, thereby accelerating wound repair and remodeling (Liu et al., 2026b).

In cartilage repair, dECM hydrogels can reproduce important features of the articular cartilage microenvironment, including avascularity, hypoxia, and high GAG content, making them promising scaffold materials for cartilage regeneration. A 2025 review reported that dECM-based repair materials may help overcome the limitations of conventional microfracture surgery, which often leads to fibrocartilage formation with suboptimal repair quality, and may better support functional hyaline cartilage regeneration and remodeling (Xu et al., 2025). A 2026 study developed photo-activatable cartilage dECM hydrogels. By introducing photosensitive groups, these materials enabled controllable patterned crosslinking and spatiotemporal regulation of local oxygen gradients, thereby better matching the gradient structure and microenvironmental characteristics of the osteochondral interface and providing an engineered tool for integrated repair of osteochondral defects (Li L. et al., 2026).

Progress has also been reported in the repair of parenchymal organs and vascular tissues. A 2026 study in Nano Today constructed a dECM-functionalized nanozyme-cell microgel platform for acute liver failure, achieving antioxidant, anti-inflammatory, and hepatocyte-regenerative effects in a rat model. The platform significantly reduced serum ALT and AST levels and improved the 7-day survival rate of model animals (Ge et al., 2026). In vascular regeneration, Wu and colleagues used decellularized vascular matrix to engineer a platelet-rich plasma-loaded tissue-engineered vascular graft in 2025. The graft suppressed early inflammation and material degradation after implantation and showed favorable long-term patency in a rabbit carotid artery replacement model (Wu et al., 2026). In addition, studies from 2024 to 2025 have shown that adipose-derived dECM hydrogels can serve as effective delivery vehicles for mesenchymal stem cells, with potential applications in wound healing and soft-tissue defect repair (Chen et al., 2025; Soltanmohammadi et al., 2025).

#### 3.3.1 Progress and controversies in dECM immunomodulatory mechanisms

Macrophage polarization is considered one of the important mechanisms by which dECM mediates tissue repair. It is generally believed that dECM can regulate the host immune microenvironment, promoting the polarization of macrophages from the pro-inflammatory (M1) to the pro-repair (M2) phenotype, thereby reducing inflammation and promoting tissue regeneration. However, in recent years, an increasing number of original studies have shown that dECM-induced M2 macrophage polarization is not a universal and stable biological effect, and its immunomodulatory capacity is influenced by multiple factors including material composition, local inflammatory status, and exogenous immune signals.

In 2022, Gvaramia and colleagues used decellularized cartilage extracellular matrix as a research model to systematically evaluate its immunomodulatory effects on human primary macrophages. The study found that cartilage dECM alone did not induce typical M2 polarization, but rather upregulated both pro-inflammatory (M1) and tissue remodeling (M2) related factors, forming a mixed M1/M2 immune phenotype and further inhibiting the migration capacity of cartilage stem/progenitor cells (CSPCs). When further combined with IL-4 loading, the material-induced inflammatory response was significantly attenuated, macrophages gradually transitioned toward an M2-like phenotype, and CSPC migration capacity was restored. The authors concluded that dECM alone is insufficient to establish a stable pro-repair immune microenvironment, and that appropriate immunomodulatory strategies are important for realizing its regenerative potential (Gvaramia et al., 2022).

Corroborating this finding, Jiang and colleagues (2025) further demonstrated under inflammatory stimulation (LPS/IFN-γ) conditions that although dECM scaffolds could promote M2 macrophage polarization to some extent, they were insufficient to sustainably suppress inflammatory responses. The research team further loaded regulatory T cell-conditioned medium (Treg-CM) onto dECM scaffolds, which significantly increased the M2/M1 ratio and effectively inhibited NF-κB signaling pathway activation, ultimately achieving superior tissue repair outcomes. This study further suggests that dECM should be viewed as a biomaterial platform with immune plasticity, and that its ultimate repair efficacy depends on synergistic regulation between the material and the local immune microenvironment, rather than the material itself being able to sustainably induce stable pro-repair immune responses (Jiang et al., 2025).

It is worth noting that the above studies do not negate the immunomodulatory effects of dECM, but rather indicate that its immunomodulation is clearly context-dependent. In the future, the design philosophy of dECM should gradually shift from “innate immunomodulation” toward “programmable immunomodulation,” achieving more precise tissue regeneration through the synergistic optimization of materials engineering and immunoengineering.

In summary, the classical understanding that “dECM naturally promotes M2 macrophage polarization” is being continuously refined by recent evidence. Available data indicate that dECM-induced immunomodulation is not a fixed M2 polarization process, but rather a dynamic immune remodeling process influenced by tissue origin, decellularization protocols, material degradation behavior, and the local inflammatory microenvironment. Therefore, future research should further establish standardized immunological evaluation systems to systematically elucidate the interaction mechanisms between dECM from different sources and host immune responses, providing a theoretical basis for the precise design and clinical translation of dECM immunomodulatory functions.

### 3.4 Summary of application landscape

From the representative advances in the three major directions of 3D bioprinting, organ-on-a-chip and organoid culture, and tissue-specific repair discussed above, the functional role of dECM has completed a paradigm shift from a “passive structural filler scaffold” to an “active functional bioplatform. “ To more clearly present the core functions and key technology pathways of each application direction, this review systematically summarizes the representative studies in Table 2. The table is primarily supported by studies discussed in detail in the preceding sections, while also incorporating additional research cases that, due to space constraints, could not be fully elaborated in the corresponding paragraphs but hold significant reference value for understanding specific technical directions, thereby providing readers with a more complete literature view. In these three scenarios, dECM plays distinct roles: in 3D bioprinting systems, it serves as the spatial architect that precisely replicates tissue morphology and constructs three-dimensional structures; in organ-on-a-chip and organoid culture systems, it serves as the signal translator that precisely reconstructs tissue-specific microenvironments and recapitulates *in vivo* cellular behaviors; in in vivo tissue injury repair, it serves as the biological commander that coordinately regulates multiple pathways and initiates endogenous regeneration. The core mechanism across all three application scenarios is highly unified: dECM is essentially a natural supramolecular assembly enriched with tissue-specific proteins, growth factor profiles, and immunomodulatory signals. The full realization of its platform application value is highly dependent on standardized preparation quality control systems and precise scenario-specific adaptation strategies.

**TABLE 2:** Representative and recent breakthrough studies in dECM applications (2024–2026).

Application area	Core function	Representative study	Key finding/Technology
3D Bioprinting	Printability-bioactivity balance	(Palierse et al., 2025)	2% w/v dECM as optimal balance point
	Unmodified native dECM printing	(Jordan et al., 2025)	Macromolecular crowding enables rapid physical crosslinking of unmodified dECM
	Liver, islet, muscle printing	(Brumberg et al., 2024; Perrier et al., 2025; Sabetkish et al., 2026)	Tissue-specific ink amplification; islet function preservation; *in vivo* validation of muscle constructs
Organ-on-a-Chip	Dynamic modeling of tumor immune microenvironment	(Li Y. et al., 2026)	5% dECM-GelMA composite enables real-time tracking of NK cell-targeted killing
	Cartilage and brain organoid chips	(Upadhyay et al., 2024; Wu et al., 2024)	Triple-component composite bioink; rat brain-derived dECM hydrogel
Tissue Repair	Multi-target myocardial infarction repair	(Mesfin et al., 2025)	Single injection activates six endogenous repair pathways
	Clinical study of hard-to-heal wounds	(Soong et al., 2025)	89-patient real-world data; 12-week closure rate 62%
	Liver failure, vascular, osteochondral repair	(Ge et al., 2026; Wu et al., 2026; Garna et al., 2026)	Multimodal synergistic therapy; PRP coating for anti-degradation; standardized cell-derived dECM

## 4 Clinical translation

Despite rapid progress in fundamental and applied dECM research, substantial barriers remain before large-scale clinical translation can be achieved. Current challenges are mainly concentrated in four areas: preparation standardization, balancing of material properties, regulatory and industrial systems, and alignment between production costs and clinical benefits.

First, the lack of unified standards for dECM preparation remains a major challenge. An ideal standardized system should include not only decellularization efficiency evaluation, such as residual DNA content (typically required to be below 50 ng/mg dry weight ECM), but also ECM composition preservation (collagen, GAGs, and key protein ratios), active factor retention levels, biomechanical properties (elastic modulus, tensile strength, and degradation behavior), sterility and endotoxin testing, and batch-to-batch consistency evaluation. In addition, for dECM from different tissue sources, tissue-specific functional evaluation indicators need to be established, such as angiogenic capacity, immunomodulatory capacity, or cell differentiation-inducing capacity. Therefore, the current challenge in dECM standardization lies not only in establishing unified production processes, but more importantly in establishing multidimensional quality control systems covering structure, composition, bioactivity, and functional output. Although current combinatorial decellularization protocols have improved preparation outcomes, differences in tissue sources, reagent batches, process parameters, and operating procedures across research groups can lead to poor batch stability and inconsistent product quality. Jin and colleagues noted in 2025 that tissue-specific dECM characterization and quality-control systems are urgently needed (Jin et al., 2025). Laude and colleagues further proposed that quality-control standards for clinical translation should not rely solely on residual DNA content. Instead, a multidimensional evaluation system is needed, incorporating GAG subtype composition, retention of key growth-factor activity, and preservation of ECM protein post-translational modifications, to provide a more comprehensive basis for assessing dECM quality (Laude et al., 2026).

Second, immunological safety and mechanical performance remain difficult to balance. Residual short DNA fragments may trigger innate immune responses through the TLR9 pathway, while alpha-Gal antigen epitopes in xenogeneic tissue-derived dECM are another important risk factor for xenogeneic immune rejection. Both issues affect the long-term *in vivo* safety of dECM (Jin et al., 2025). At the same time, decellularization removes cellular structures and some functional components, leaving dECM with mechanical properties that are often weaker than those of native tissues. This limitation is particularly relevant for load-bearing tissues such as bone, cartilage, and blood vessels. Chemical crosslinking methods, including glutaraldehyde treatment, can enhance mechanical strength, but they may also introduce cytotoxicity, alter degradation behavior, and reduce bioactivity. Notably, several emerging strategies reviewed in the preceding sections have made promising explorations in this direction. Macromolecular crowding strategy accelerates dECM self-assembly through physical volume exclusion effects, achieving printable mechanical strength without introducing any exogenous crosslinkers (Jordan et al., 2025). Photo-crosslinkable modification systems endow dECM with light-controllable crosslinking capability through methacrylation, avoiding the cytotoxicity of traditional crosslinkers such as glutaraldehyde while achieving digitally tunable crosslinking density and mechanical properties (Kim M. et al., 2025; Kafili et al., 2026). Furthermore, granular dECM hydrogels form macroporous interconnected networks through microgel particle packing, and their mechanical behavior can be modularly programmed through particle size, packing density, and inter-particle crosslinking degree, providing a “top-down” design paradigm for mechanical matching of load-bearing tissues (Feng et al., 2025). The above advances indicate that “non-chemical crosslinking-based mechanical enhancement” has moved from concept validation to a stage where multiple technological pathways coexist. However, core issues such as long-term *in vivo* stability and the spatiotemporal synchronization of degradation and mechanical attenuation for each pathway still require systematic investigation. Achieving precise mechanical matching between dECM and host tissues in a minimally modified, native matrix system remains an important engineering challenge.

Third, regulatory definitions remain unclear, and the industrial system is still immature. No globally unified regulatory classification currently exists for dECM products. Different countries primarily implement classification management based on product source, processing method, and intended use, leading to different approval pathways for similar products under different regulatory systems (Jin et al., 2025). Currently, the FDA has not established an independent regulatory category for dECM, instead adopting a risk-based classification approach. For human-derived ECM products that meet the criteria of minimal manipulation and homologous use, they are regulated as Human Cells, Tissues, and Cellular and Tissue-Based Products (HCT/Ps) under 21 CFR Part 1271. If products undergo more than minimal manipulation or have pharmacological, immunological, or metabolic effects, they may be subject to premarket review as medical devices, biologics, or combination products, with different review pathways such as 510(k), PMA, or BLA applicable. At present, several ECM products have been authorized for clinical use through the 510(k) clearance pathway: OASIS® Wound Matrix (small intestinal submucosa, K061711), Strattice™ Reconstructive Tissue Matrix (porcine dermis, K070560), and InnovaMatrix® AC (porcine placenta, K210580) have all received FDA 510(k) clearance as medical devices. This diversity in regulatory pathways reflects the FDA’s risk-based classification approach to ECM-based products.

Fourth, production costs and clinical benefits remain insufficiently aligned. Under Good Manufacturing Practice (GMP) conditions, dECM preparation is technically complex and quality control is costly, resulting in production costs that are generally higher than those of conventional synthetic biomaterials. At present, many dECM-based products are mainly used for refractory or salvage clinical indications in which conventional treatments have failed, limiting their application scope. For dECM to move from niche salvage therapy toward first-line clinical use, future work must reduce production costs through end-to-end process optimization, workflow simplification, and scalable manufacturing. At the same time, large-cohort clinical studies with long-term follow-up are needed to validate therapeutic benefits and cost-effectiveness.

## 5 Conclusion and future perspectives

dECM has moved beyond the single structural function of conventional biological scaffolds and is undergoing a transition from a passive repair scaffold to a multifunctional biological regulation platform. Over the past 2 decades, research has driven dECM from “cell-free natural scaffolds” to bioactive material systems with tissue-specific functions, and the rapid developments since 2020 have further accelerated this paradigm shift. In recent years, combinatorial decellularization strategies, intelligent preparation workflows, and multidimensional quality evaluation systems have continuously improved, enhancing the reproducibility and functional preservation of dECM preparation processes. Bioprinting, macromolecular crowding, self-assembly regulation, and photo-crosslinking modification strategies have further expanded the application boundaries of dECM in complex tissue construction. Proteomics, multi-omics analysis, and advanced characterization technologies have driven researchers from traditional “composition description” into the “functional profiling” stage, providing new theoretical foundations for understanding the tissue-specific mechanisms of dECM action. Furthermore, preclinical and clinical studies in areas such as myocardial repair, chronic wound treatment, cartilage regeneration, and organ engineering have demonstrated that dECM can achieve complex tissue repair by modulating the immune microenvironment, promoting angiogenesis, and coordinating multicellular behaviors, exhibiting unique regenerative potential distinct from traditional biomaterials.

However, multiple challenges remain for dECM to move from experimental research to large-scale clinical application. First, differences in tissue sources, decellularization protocols, and processing methods lead to significant variations in dECM composition and bioactivity, and there is currently a lack of unified preparation standards and quality evaluation systems. Second, although dECM has good biocompatibility, its immunomodulatory effects are highly context-dependent, and how to achieve stable and predictable immune repair responses still requires further elucidation. In addition, native dECM materials still have limitations in mechanical properties, processing stability, and long-term functional maintenance, restricting their further application in complex load-bearing tissues and solid organ engineering. Therefore, the core goal of future dECM research is not simply to improve material performance, but to achieve precise matching among material design, manufacturing processes, and host responses while maintaining native biological activity.

Looking ahead, the dECM field is likely to develop along four major directions: precision construction, functional organ engineering, programmable immunomodulation, and intelligent manufacturing.

First, patient-specific precision dECM construction will become an important direction for personalized regenerative medicine. Combining patient-derived tissues, medical image reconstruction, computational modeling, and 3D bioprinting technologies, it is expected to achieve dECM scaffold design with individual structural matching and functional requirement matching. In the future, the integration of automated decellularization workflows and digital manufacturing systems will further drive dECM from the current “universal biomaterial” toward “patient-specific therapeutic platforms. “ However, limitations in patient-derived material availability, preparation timelines, and batch consistency remain challenges to be addressed for precision applications.

Second, whole-organ decellularization and recellularization technologies will continue to advance organ engineering from structural reconstruction toward functional maturation. Whole-organ dECM engineering is not a new concept proposed in recent years, but rather a continuous evolution based on more than a decade of research in organ perfusion decellularization and recellularization. Early studies primarily demonstrated that native ECM scaffolds can preserve complex three-dimensional anatomical structures, vascular networks, and tissue-specific microenvironments, providing a foundation for subsequent cell reconstruction. In 2010, Petersen and colleagues used perfusion decellularization technology to construct rat lung dECM scaffolds and achieved partially functional lung tissue reconstruction through re-implantation of alveolar epithelial cells and vascular endothelial cells, for the first time demonstrating that organ-level dECM scaffolds can be used for *in vivo* functional tissue engineering, laying an important foundation for whole-organ regeneration (Petersen et al., 2010). Subsequently, research focus gradually shifted from “obtaining complete decellularized scaffolds” toward “improving recellularization efficiency and functional maturation. “ In 2015, the Ott team further addressed the key bottleneck of insufficient vascular network reconstruction during lung dECM scaffold recellularization, systematically optimizing pulmonary vascular engineering strategies for rat and human lung decellularized scaffolds. Through endothelial cell perfusion and vascularization regulation, they improved the reconstruction capability of the pulmonary vascular system, advancing lung dECM engineering from the structural preservation stage to the functional vascularization stage (Ren et al., 2015). However, early whole-organ recellularization systems still faced challenges such as low cell entry efficiency, insufficient deep tissue distribution, and difficulty in long-term functional maintenance. In recent years, researchers have begun addressing these limitations from the perspectives of bioreactor parameter optimization, mechanical microenvironment regulation, and multi-cell co-culture. For example, Ahmadipour and colleagues reported in 2021 that increasing ventilation frequency in the culture system improved lung dECM scaffold recellularization efficiency. This study found that enhanced dynamic ventilation conditions promoted the migration and spatial distribution of lung epithelial cells in decellularized lung scaffolds, increasing alveolar region cell coverage, suggesting that mechanical microenvironment regulation not only affects cell survival but also participates in organ-level tissue reconstruction and functional maturation processes (Ahmadipour et al., 2021). In the field of kidney dECM engineering, Choi and colleagues addressed the issue of insufficient vascularization during whole-kidney ECM scaffold recellularization in 2022, proposing a strategy combining optimized perfusion flow rate with multi-cell co-culture. By co-seeding mesenchymal stem cells (MSCs) and glomerulus endothelial cells (GECs) into whole-kidney ECM scaffolds, they systematically evaluated cell distribution and endothelialization under different perfusion conditions. The results showed that optimized perfusion flow rates improved cell adhesion and migration within the complex three-dimensional kidney structure, while MSC and GEC co-culture further promoted endothelial cell colonization and vascular-like structure formation, enhancing the vascularization level of kidney dECM scaffolds. This study demonstrated that the combination of dynamic mechanical environment regulation and multi-cell interactions is an important development direction for improving the efficiency of functional reconstruction in complex organ dECM engineering (Choi et al., 2022). Addressing the lack of long-term dynamic evaluation methods during recellularization of metabolic organs such as the liver, Sassi and colleagues developed a perfusion bioreactor for engineered liver tissue construction in 2021, achieving longitudinal continuous monitoring of bioengineered liver tissue culture processes. This system enables real-time tracking of construct changes in dynamic perfusion environments, providing a new technological platform for evaluating cell status, optimizing culture parameters, and analyzing tissue maturation processes. This study promotes liver dECM engineering from traditional endpoint detection mode after culture completion toward whole-process dynamic monitoring and quality control mode, providing important tools for future large-scale, standardized organ engineering construction (Sassi et al., 2021). Therefore, in recent years, the focus of whole-organ dECM recellularization technology development has shifted from early concerns about “whether cell implantation can be achieved” toward how to achieve more stable and mature organ-level functional recovery through mechanical microenvironment regulation, multi-cell co-culture, and real-time process monitoring. At present, stable vascularization at large animal scale, post-transplantation host integration, and long-term safety evaluation remain core challenges limiting the clinical translation of this technology. Nevertheless, the future of whole-organ decellularization/recellularization should not be considered as a competing strategy against emerging biofabrication approaches such as 3D bioprinting. Native organ-derived dECM scaffolds provide unmatched advantages in preserving hierarchical vascular networks, tissue-specific matrix composition, and endogenous spatial cues; however, their limited flexibility in precisely positioning multiple cell populations remains a major challenge. In contrast, 3D bioprinting enables programmable cellular organization but currently lacks the resolution and biological complexity required to fully reconstruct native organ microarchitecture. Therefore, future advances may rely on hybrid strategies that combine vascularized native dECM frameworks with spatially controlled bioprinting technologies. Beyond whole-organ replacement, dECM scaffolds are also emerging as functional biomaterial platforms for regenerative medicine. For example, Citro et al. demonstrated that decellularized lung ECM could serve as a prevascularized scaffold to support pancreatic islet implantation, highlighting a new application paradigm in which dECM provides a vascular niche rather than directly replacing an entire organ (Citro et al., 2023). These developments suggest that the future impact of whole-organ dECM technologies may extend from organ reconstruction toward multifunctional platforms for cell-based therapies.

Third, dECM immunomodulation will gradually shift from reducing immunogenicity toward actively programming immune repair. Past dECM research primarily focused on cellular residue removal and host immune rejection reduction, but increasing evidence in recent years indicates that dECM degradation products, matrix protein fragments, and tissue-specific signals can actively influence macrophage behavior, inflammatory conversion, and tissue remodeling processes. Future research needs to further elucidate the causal relationships among dECM composition, degradation kinetics, and immune cell responses, and combine cytokine delivery, immunomodulatory molecule loading, and temporal control strategies to achieve the transition from empirically promoting repair toward precision immunoengineering regulation.

Fourth, intelligent manufacturing systems will become an important foundation for promoting dECM industrialization. The main challenges currently facing dECM product commercialization include source variation, batch fluctuation, insufficient quality evaluation standards, and high production costs. In the future, through online multi-index monitoring technologies, multi-omics quality evaluation, artificial intelligence-assisted process optimization, and closed-loop GMP production systems, it is expected to achieve dynamic control of decellularization processes and product performance prediction, thereby improving the consistency and clinical acceptability of dECM preparation. At the same time, regulatory systems also need further improvement, establishing comprehensive evaluation standards covering residual DNA, ECM composition, growth factor retention, biomechanical properties, immune safety, and long-term stability, providing a basis for standardized industrial development.

Overall, the development of dECM is undergoing a profound transformation from “natural scaffold utilization” toward “designable, regulatable, and manufacturable functional regenerative platforms. “ In the future, key breakthroughs in this field will no longer rely on single material performance improvement, but on the synergistic development of fundamental mechanism research, engineering technology innovation, and regulatory system construction. Only by establishing standardized preparation systems, clarifying immunomodulatory mechanisms, and achieving scalable quality control can dECM truly complete the translation from laboratory research to clinical therapeutic platforms.
