# Correction: From biological scaffold to multifunctional bioplatform: research progress and applications of decellularized extracellular matrix

**DOI:** 10.3389/fbioe.2026.2013027

**Published:** 2026-10-16

**Authors:** Xin Li, Mingzhu Wang, Pei Du

**Affiliations:** 1 Department of Gynaecology and Obstetrics, The Affiliated Panyu Central Hospital, Guangzhou Medical University, Guangzhou, Guangdong, China; 2 Institute of Insect Science and Technology & School of Life Sciences, South China Normal University, Guangzhou, China

**Keywords:** 3D bioprinting, bioinks, decellularized extracellular matrix (dECM), organ on a chip, regenerative medicine, tissue engineering

The reference for Liu, X., Zhao, T., Mu, Y., Feng, W., Lyu, C., Zhang, Z., et al. (2026b). Acellular dermal matrix hydrogel promotes skin wound healing in rats. Chin. J. Tissue Eng. Res. 30, 395–403. doi: 10.12307/2025.893 was erroneously written as (刘 et al., 2024). It should be (Liu et al., 2026b).

The original version of this article has been updated.

